# Influence of Microbes on Neutrophil Life and Death

**DOI:** 10.3389/fcimb.2017.00159

**Published:** 2017-05-01

**Authors:** Scott D. Kobayashi, Natalia Malachowa, Frank R. DeLeo

**Affiliations:** Laboratory of Bacteriology, Rocky Mountain Laboratories, National Institute of Allergy and Infectious Diseases, National Institutes of HealthHamilton, MT, USA

**Keywords:** phagocytosis, host defense, apoptosis, necroptosis, programmed cell death, necrosis

## Abstract

Neutrophils are the most abundant leukocyte in humans and they are among the first white cells recruited to infected tissues. These leukocytes are essential for the innate immune response to bacteria and fungi. Inasmuch as neutrophils produce or contain potent microbicides that can be toxic to the host, neutrophil turnover and homeostasis is a highly regulated process that prevents unintended host tissue damage. Indeed, constitutive neutrophil apoptosis and subsequent removal of these cells by mononuclear phagocytes is a primary means by which neutrophil homeostasis is maintained in healthy individuals. Processes that alter normal neutrophil turnover and removal of effete cells can lead to host tissue damage and disease. The interaction of neutrophils with microbes and molecules produced by microbes often alters neutrophil turnover. The ability of microbes to alter the fate of neutrophils is highly varied, can be microbe-specific, and ranges from prolonging the neutrophil lifespan to causing rapid neutrophil lysis after phagocytosis. Here we provide a brief overview of these processes and their associated impact on innate host defense.

## Introduction

Neutrophils are the primary cellular defense against infections caused by bacteria and fungi. These phagocytes are the most numerous leukocyte in humans and are mobilized rapidly from the bloodstream and/or bone marrow reserves to infected tissues. At sites of infection, neutrophils phagocytose microbes, which in turn are killed by reactive oxygen species (ROS) and antimicrobial peptides and proteases (reviewed in Nauseef, 2007). Many of these microbicidal molecules are also cytotoxic to host tissues and can contribute to the pathogenesis of infection (Weiss, 1989; Diep et al., 2010). Therefore, it is not surprising that multiple mechanisms are employed by the host to limit or prevent damage to host tissues and unintended inflammation. For example, neutrophil activation, which includes the production of neutrophil ROS and mobilization of antimicrobial peptides/proteins, is a highly regulated process. From a broader perspective, the regulation of neutrophil production and turnover are processes that are required to maintain immune system homeostasis and ultimately health of the host.

There is enormous daily production of neutrophils in healthy humans. Approximately 60% of the cells in bone marrow are granulocytes and granulocyte precursors, the vast majority of which develop into neutrophils. Neutrophils mature in bone marrow as post-mitotic precursor cells for approximately 5–6 days, at which point they are released into circulation as mature neutrophils (Fliedner et al., 1964; Dancey et al., 1976; Macallan et al., 1998). Remarkably, ~50–70% of leukocytes in circulation are neutrophils. During steady-state conditions, these cells typically circulate in the bloodstream for ~12–18 h (the circulating granulocyte pool, reviewed by Tak et al., 2013), although a recent study suggests a longer time in circulation (Pillay et al., 2010). Neutrophils then enter tissues such as spleen, liver or bone marrow (Summers et al., 2010). These cells, which comprised the marginated granulocyte pool, reside in tissues for 1–2 days and ultimately undergo spontaneous apoptosis and are removed by mononuclear phagocytes. During steady state conditions, ~10^11^ neutrophils turn over per day in a healthy human adult (Athens et al., 1961; Cartwright et al., 1964). Thus, spontaneous or constitutive neutrophil apoptosis and subsequent removal by mononuclear phagocytes is critical for maintenance of immune system homeostasis. Neutrophil apoptosis is also an important means to eliminate effete or damaged cells during disease conditions and promote the resolution of inflammatory responses (Bratton and Henson, 2011).

Significant progress has been made toward understanding neutrophil apoptosis over the past few decades. Landmark studies by Savill et al. reported that aged neutrophils undergo programmed cell death (apoptosis), and this process is accompanied by nuclear chromatin condensation, DNA fragmentation, and cytoplasmic vacuolation (Savill et al., 1989). Apoptotic neutrophils in those studies remained intact for at least 24 h and there was no release of granule enzymes. Importantly, there is a direct correlation between neutrophil apoptosis and their nonphlogistic uptake by macrophages—a process now known as efferocytosis (Newman et al., 1982; Savill et al., 1989, 1990). Subsequent work by Whyte and colleagues revealed that apoptosis of neutrophils is accompanied by decreased cell function, which includes reduced antimicrobial and proinflammatory capacities (Haslett et al., 1991; Whyte et al., 1993). These studies were early descriptions of spontaneous neutrophil apoptosis and their removal by macrophages. The findings led to the notion that removal of effete neutrophils at sites of inflammation limits damage to host tissues and is essential for the resolution of inflammation (Grigg et al., 1991). From a mechanism standpoint, neutrophil apoptosis can proceed by way of an intrinsic pathway, which involves permeabilization of mitochondria and release of proapoptosis molecules into the cytosol (Maianski et al., 2004), or an extrinsic pathway, triggered by death receptors such as FAS (CD95) or the TNF receptor (Geering et al., 2013). For more detail about these mechanisms, we refer the reader to reviews on the topic (Simon, 2003; Geering et al., 2013; Green and Llambi, 2015; Wallach et al., 2016).

Inasmuch as there is sustained need for neutrophils during microbial infections and other inflammatory processes, it is perhaps not unexpected that proinflammatory molecules, whether host or pathogen-derived, can delay spontaneous apoptosis and prolong the neutrophil lifespan (Colotta et al., 1992; Lee et al., 1993). For example, proinflammatory cytokines and bacterial molecules, including human recombinant C5a, granulocyte-colony stimulating factor (G-CSF), granulocyte-macrophage colony-stimulating factor (GM-CSF), interleukin-1β, interleukin-6, interferon-γ, lipopolysaccharide, or tumor necrosis factor, delay human neutrophil apoptosis *in vitro* (Colotta et al., 1992; Lee et al., 1993). It is now known that many such molecules alter neutrophil survival (Geering et al., 2013), and some of these molecules, such as G-CSF and GM-CSF, increase production of neutrophils (Nauseef and Borregaard, 2014). Proinflammatory molecules that prolong neutrophil survival typically prime neutrophils for enhanced function.

The host microenvironment can also have an important impact on neutrophil survival and function, especially in the context of sites of inflammation and infection. Notably, Chilvers and colleagues found that hypoxia inhibits neutrophil apoptosis significantly (Hannah et al., 1995), and that this phenomenon is regulated by hypoxia-inducible factor-1 alpha (HIF-1α) (Walmsley et al., 2005). Despite the increased lifespan, neutrophils in hypoxic conditions have reduced ability to kill *S. aureus* (McGovern et al., 2011; Lodge et al., 2017), a defect linked to the inability of neutrophils to produce NADPH oxidase-dependent reactive oxygen species (McGovern et al., 2011).

Direct interaction with intact microbes, including bacteria, fungi, parasites, and viruses, also influences neutrophil survival. The outcome of these interactions ranges from delay of apoptosis and prolonged neutrophil lifespan to rapid neutrophil lysis after phagocytosis. Although progress has been made, specific mechanisms for many of these processes remain incompletely determined. Here we use selected microbe-neutrophil interactions to review in these processes in brief.

## Bacteria

The ability of phagocytes to ingest and subsequently kill invading microbial pathogens is requisite for maintenance of host health. Neutrophils are recruited to infection sites through a combination of host proinflammatory cytokines and chemokines and microbe-derived chemotactic factors. In addition to host proinflammatory factors, shed bacterial surface molecules such as LPS and LTA, and secreted toxins (e.g., *Escherichia coli* verotoxin, Shiga toxins, and phenol-soluble modulins) delay neutrophil spontaneous apoptosis, effectively increasing neutrophil numbers during early stages of inflammation and thus providing ample opportunity for clearance of invading pathogens (Sabroe et al., 2003; Lotz et al., 2004). Phagocyte recognition of microbial pathogens is mediated by receptors present on the outer surface of the host cell membrane. The two primary types of neutrophil receptors that are used to recognize microorganisms are pathogen recognition receptors (PRRs), which directly recognize microbial-derived structures, and opsonic receptors that recognize host proteins deposited on the microbial surface. Although PRRs are critical for immune surveillance and contribute to enhanced phagocyte effector functions, phagocytosis is most efficient in the presence of opsonins–soluble host molecules that promote uptake–of which specific IgG and complement are the major constituents. Neutrophil recognition of antibody and/or complement deposited on microbial surfaces directly mediates phagocytosis. The process of phagocytosis induces apoptosis in human neutrophils (also known as phagocytosis-induced cell death—PICD) (reviewed by Kobayashi and DeLeo, 2009), and the induction of apoptosis is intimately linked to production of ROS following activation (Coxon et al., 1996; Kobayashi et al., 2002). *E. coli* was one of the first bacterial pathogens identified to induce neutrophil apoptosis after phagocytosis (Watson et al., 1996b). This study was followed by reports of taxonomically diverse bacterial pathogens such as *Borrelia hermsii, Streptococcus pneumoniae, Mycobacterium tuberculosis*, and *Listeria monocytogenes* that similarly accelerate neutrophil apoptosis (reviewed by Kennedy and DeLeo, 2009). It is of note that PICD occurs in the face of prosurvival signals imparted by proinflammatory cytokines or bacteria-derived factors (Watson et al., 1996a). Ultimately, these effete neutrophils are removed through efferocytosis, thus promoting termination of the inflammatory process.

Although PICD is a component of normal antimicrobial host defense, bacterial pathogens are capable of altering the normal course of PICD as a mechanism of virulence. In general, the relatively short neutrophil lifespan is not amenable to long-term survival strategies employed by many intracellular bacterial pathogens and there are few pathogens with tropism for neutrophils. *Anaplasma phagocytophilum*, the etiologic agent of human granulocytic anaplasmosis, is one of the few pathogens with neutrophil tropism and was the first bacterial pathogen reported to delay human neutrophil apoptosis (Yoshiie et al., 2000). *A. phagocytophilum* is capable of manipulating NADPH oxidase assembly—a feature that is shared with another intracellular bacterial pathogen, *Francisella tularensis* (McCaffrey et al., 2010)—and the disruption of ROS production facilitates pathogen survival and replication within neutrophils. In addition, *F. tularensis* inhibits neutrophil spontaneous apoptosis through activation of PI3K/Akt and ERK1/2 MAPKK survival pathways (McCracken et al., 2016). *Chlamydia pneumoniae* and *Neisseria gonorrhoeae* have also been reported to delay neutrophil apoptosis (van Zandbergen et al., 2004; Simons et al., 2006; Chen and Seifert, 2011). Thus, some bacteria—and those that are predominantly intracellular pathogens—can extend the neutrophil lifespan by disrupting the normal process of spontaneous apoptosis and/or PICD. On the other end of the spectrum, some pathogens such as *Streptococcus pyogenes* are capable of altering neutrophil fate after phagocytosis by promoting rapid cell lysis and/or accelerating apoptosis to the point of secondary necrosis (Kobayashi et al., 2003). Consistent with the idea that neutrophil lysis exacerbates acute inflammation and facilitates pathogenesis, *S. pyogenes* often presents clinically with highly necrotic lesions. Moreover, some highly virulent strains of *Staphylococcus aureus* promote rapid neutrophil lysis after phagocytosis and recent evidence suggests that the process occurs by programmed necrosis or necroptosis (Greenlee-Wacker et al., 2014). Necroptosis is a proinflammatory form of cell death dependent on receptor interacting protein-1 kinase and leads to necrotic cell lysis. Inasmuch as neutrophil lysis is generally unfavorable to host health, bacteria-induced lysis should be considered an important facet of bacterial pathogenesis.

**Figure 1:**
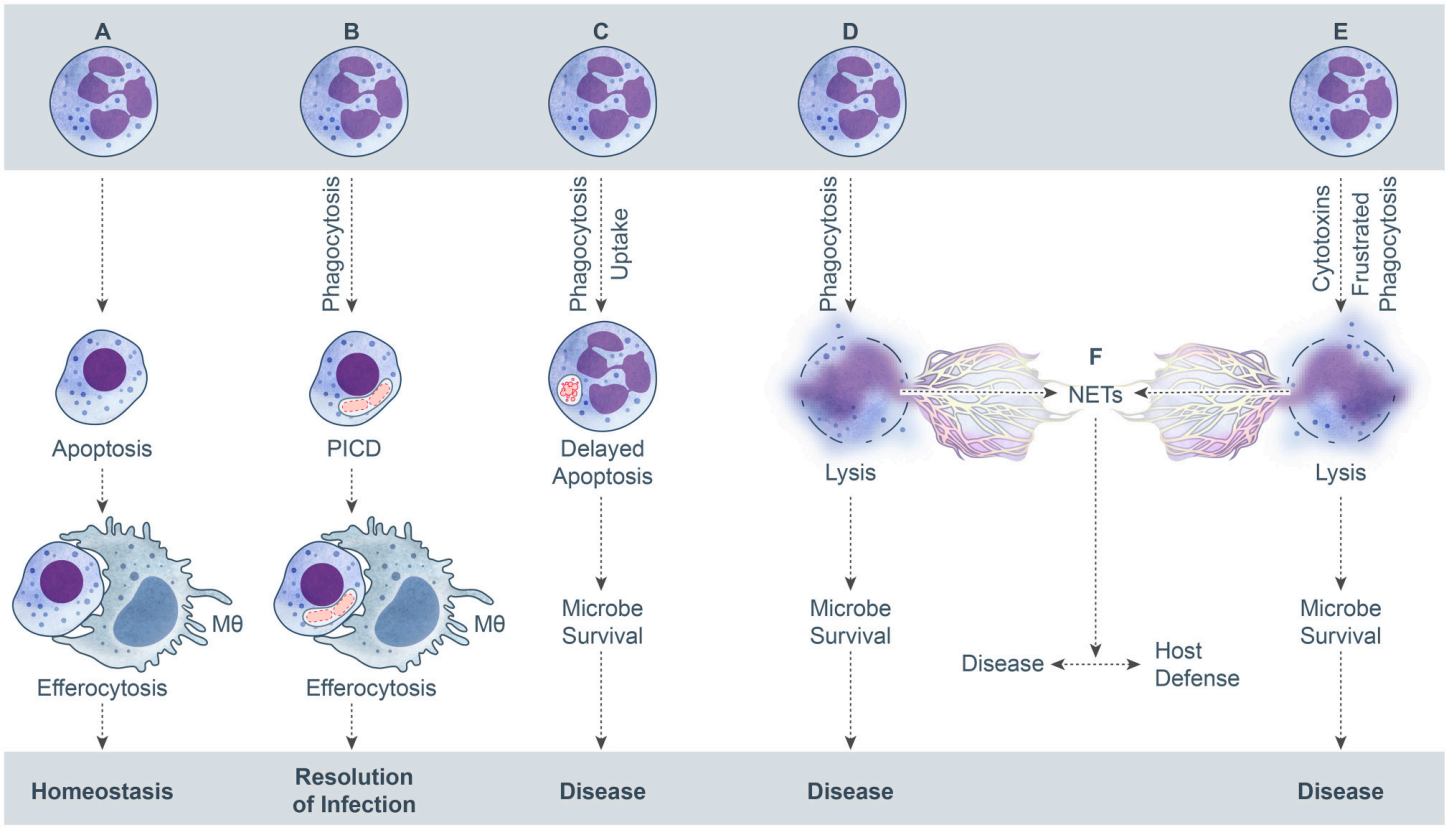
**Possible outcomes of neutrophil-microbe interactions. (A)** Spontaneous neutrophil apoptosis and removal by a macrophage. **(B)** Phagocytosis-induced cell death (PICD) and subsequent macrophage phagocytosis of the apoptotic neutrophil. **(C)** Neutrophil uptake of a microbe—by phagocytosis or some other mechanism—that leads to delay of neutrophil apoptosis as a means to promote microbe survival. **(D)** Neutrophil lysis after phagocytosis. Cytolysis can be programmed, e.g., necroptosis, or caused by direct damage. **(E)** Neutrophil lysis caused by cytolytic toxins, pore-forming agents, physical injury, or frustrated phagocytosis. **(F)**. Formation of neutrophil extracellular traps (NETs) during neutrophil lysis.

Neutrophil extracellular traps (NETs) are elicited by many conditions, including interaction with microbial pathogens. NETs can form from viable cells or by cytolysis, and are characterized by filamentous web-like structures that consist of extruded DNA that are ornamented with neutrophil granule enzymes and histones (Brinkmann et al., 2004; Yousefi et al., 2009). Neutrophils that form NETs by a cytolytic process were originally proposed to undergo a special form of death known as NETosis (Fuchs et al., 2007). However, the notion that NETosis is distinct from necrotic cell death has been brought into question recently (Malachowa et al., 2013, 2016; Nauseef and Kubes, 2016; Yousefi and Simon, 2016). For the purposes of this review, we consider formation of NETs that occurs concomitant with neutrophil lysis as necrotic cell death (Figure 1). A large number of bacterial pathogens have been associated with NET formation, and although some pathogens are reported as killed by these structures (Lu et al., 2012), neutrophil lysis clearly has deleterious effects on host tissue (Henson and Johnston, 1987; Weiss, 1989).

## Fungi

Neutrophils are critical to host defense against fungal pathogens, as evidenced by increased susceptibility of patients with congenital or acquired neutrophil deficiencies to fungal infections. For example, it is well documented that individuals with chronic granulomatous disease—a genetic deficiency in NADPH oxidase—have increased incidence of infections caused by *Aspergillus* (Marciano et al., 2015). In addition, neutrophil defects and deficiencies are associated with increased susceptibility to *Candida* infections, including leukocyte adhesion deficiency and severe congenital neutropenia (reviewed by Wang et al., 2016). Although fungi are ubiquitous, there are a limited number of primary fungal pathogens that cause infection in otherwise healthy individuals—namely *Histoplasma capsulatum, Coccidioides immitis, Paracoccidiodes brasiliensis*, and *Blastomyces dermatitidis*. Compared to bacteria, fungi present a unique challenge to neutrophil killing in that they are eukaryotes and structure and morphology can vary widely from single-cell blactoconidia (e.g., 2-4-micron *H. capsulatum* yeast) to elongated hyphae or large sporulating structures such as the *Coccidioides immitis* spherule (~10–200 microns). Particle size is an additional and important consideration for efficient neutrophil phagocytosis. Consistent with the opportunistic lifestyle, there are numerous reports indicating that fungal pathogens such as *Candida albicans* are readily ingested and killed by neutrophils (Wozniok et al., 2008; Gazendam et al., 2014; Kaloriti et al., 2014) and induce apoptosis consistent with PICD (Rotstein et al., 2000). Similarly, neutrophils have been shown to target and kill *Aspergillus fumigatus* hyphae (Leal et al., 2012) and conidia (Gazendam et al., 2016) and induce neutrophil apoptosis (Robinet et al., 2014). By contrast, it is widely reported that primary fungal pathogens, including *B. dermatitidis* (Brummer and Stevens, 1982) and *H. capsulatum* (Newman et al., 1993), are significantly more resistant to killing by neutrophils (Schaffner et al., 1986). Although there are limited studies on the impact of fungal infection on neutrophil fate, *H. capsulatum* has been reported to inhibit neutrophil apoptosis and downregulate Mac-1 expression *in vitro* (Medeiros et al., 2002). Moreover, limited numbers of apoptotic neutrophils are observed in lungs of mice infected experimentally with *H. capsulatum* (Allen and Deepe, 2005).

As mentioned above, fungi produce several morphologically diverse structures and many of these features are linked to the ability to exist as both saprophytes and human pathogens. For example, many of the primary fungal pathogens are dimorphic and produce hyphae predominantly as saprophytes, and convert to a single cell state in the host. On the other hand, other pathogens such as *Aspergillus* spp. are inhaled as spores and germinate into hyphal cells within the host. In addition, *C. albicans* is characterized by the ability to produce yeast, hyphae, and pseudohyphae in host tissue. Host phagocytes are therefore confronted with a variety of fungal structures during pathogenesis. Neutrophils have an impressive capacity for phagocytosis and are capable of expanding their surface area of up to 300% of their resting area (Ting-Beall et al., 1993). That said, the ability of these phagocytes to ingest particles >10 microns is significantly diminished (Herant et al., 2006), which presents a unique challenge for neutrophil removal and killing of larger fungal structures. It has long been known that the inability of neutrophils to ingest large particles initiates a process often referred to as “frustrated phagocytosis” whereby granules are exocytosed from activated neutrophils and their contents released extracellularly (Henson, 1971; Wright and Gallin, 1979; Boyles and Bainton, 1981). Although unregulated (or excessive) extracellular release of neutrophil granule contents is detrimental to host tissues (Henson and Johnston, 1987), the combination of ROS and granule constituents are sufficient to kill both *C. albicans* (Christin et al., 1997) and *A. fumigatus* hyphae (Leal et al., 2012). The fate of the activated neutrophils in the fungal inflammatory milieu is not clear. However, there are numerous reports that describe the ability of fungi—almost exclusively opportunistic pathogens—to induce NETs. The role of NETs in host defense against fungi—if any—is unclear as some studies report that NETs have fungicidal activity (Urban et al., 2006; Branzk et al., 2014) whereas others suggest they do not (Menegazzi et al., 2012; Gazendam et al., 2016). Regardless, it is clear that neutrophils are highly effective at killing opportunistic fungal pathogens and are perhaps exploited more readily by fungi that cause primary disease.

## Parasites

In contrast to the enhanced knowledge of the role played by neutrophils in host defense against bacteria, our understanding of neutrophil interactions with parasitic protozoans and/or helminth parasites has been slower to evolve. Parasitic pathogens present unique challenges to neutrophils. For example, some parasites are sequestered by- or reside within non-neutrophil effector and destination cells. In addition, parasites such as amoebas and helminths are very large microbes, and such large size is prohibitive to phagocytosis. Nonetheless, parasites often produce chemoattractants that interact with multiple neutrophil receptors and can result in activation of either pro- or anti-inflammatory signals (Smith et al., 2005; Wenzel and van Zandbergen, 2009). As an example, *Leishmania* chemotactic factor (LCF) works through the lipoxin A4 receptor (ALX or FPLR-1) that paradoxically induces anti-inflammatory signals while simultaneously accelerating the rate of phagocytosis of parasites by neutrophils (Wenzel and van Zandbergen, 2009). By contrast, *Strongyloides stercoralis* and *Brugia malaya* larvae induce neutrophil chemotaxis through the CXCR2 receptor and this process results in the initiation of pro-inflammatory signals (Ramirez et al., 2006; O'Connell et al., 2011).

For certain intracellular parasites, the presence of neutrophils at the site of infection is beneficial for pathogen dissemination to target cells and/or organs, and plays a crucial role in perpetuating disease. The interaction of *Leishmania* spp. with neutrophils provides one of the best characterized examples of exploitation of the neutrophil lifespan by a pathogen. It is well-known that phagocytosis of apoptotic cells by mononuclear phagocytes is nonphlogistic, and the process triggers production of molecules that dampen the inflammatory response and decreases secretion of proinflammatory molecules (Voll et al., 1997). During infection with *Leishmania major*, a relatively high percentage (up to 50%) of promastigotes becomes apoptotic (van Zandbergen et al., 2006). As with macrophage phagocytosis of apoptotic host cells, the interaction of apoptotic promastigotes with neutrophils causes secretion of anti-inflammatory molecules such as TGF-β and suppression of pro-inflammatory TNFα (van Zandbergen et al., 2006). Although phagocytosis of apoptotic and non-apoptotic promastigotes by neutrophils is similar, the immune dampening effect of the apoptotic promastigotes allows some of the viable/non-apoptotic parasites to survive within neutrophils (van Zandbergen et al., 2006). Ingested *Leishmania* prolongs the neutrophil lifespan for up to 24 h by up-regulating phosphorylation of ERK1/2 pathway and decreasing caspase-3 activity (Aga et al., 2002; Sarkar et al., 2013). Ultimately, neutrophils containing *Leishmania* undergo apoptosis and are ingested by macrophages, which in turn become infected with the parasite. This “Trojan horse” style entry of parasite to its final destination–the macrophage–prevents activation of macrophages and a strong immune response (Aga et al., 2002; van Zandbergen et al., 2006).

In addition to hijacking host immune cells, parasitic pathogens can influence neutrophil maturation and subsequently impair microbicidal potential. For example, a higher percentage of immature neutrophils (band cells) with impaired phagocytic capacity and ROS production has been reported in blood samples of patients with visceral leishmaniasis (Yizengaw et al., 2016) or individuals in the acute phase of *P. falciparum* and *P. vivax* infections (Lima-Junior et al., 2012). In the latter, impaired neutrophil development was linked to release of heme during parasite-induced lysis of red blood cells, which induces heme oxygenase-1 (HO-1) that consequently affects development of myeloid progenitor cells (Cunnington et al., 2011, 2012). A high percentage of immature neutrophils increases host susceptibility to secondary infections (Cunnington et al., 2012). Moreover, parasites from mastigophora, sarcodina and sporozoa phyla as well as helminths have been shown to trigger NET formation *in vivo* and/or *in vitro* (Baker et al., 2008; Abi Abdallah et al., 2012; Rochael et al., 2015; Ventura-Juarez et al., 2016). Although there seems to be an agreement that these structures trap microbes (Hermosilla et al., 2014), data that bear on the ability of NETs to kill parasites is at variance (Guimaraes-Costa et al., 2009; Hermosilla et al., 2014; McCoy et al., 2017). Regardless of the outcome for the microbe, release of cytotoxic neutrophil contents onto surrounding tissues either by degranulation, lysis, or NET formation can be detrimental to the host (Chuah et al., 2013; Hurrell et al., 2015).

## Viruses

Viruses comprise a very diverse group of pathogens. They differ by shape, size, or type of nucleic acid that encodes their genetic information, but ultimately all viruses require a living cell in which to replicate. Following entry into a host cell, viruses hijack host machinery and force the cell to create multiple copies of the virus. Inasmuch as neutrophils are terminally differentiated, short-lived immune cells, it is debatable whether viruses can efficiently infect and proliferate in these cells. Nonetheless, numerous viruses are internalized by PMNs during infection and some, such as West Nile virus (Bai et al., 2010) and viruses that belong to the herpes virus family, are reported to replicate within neutrophils (Larochelle et al., 1998; Skarman et al., 2006; Royer et al., 2015). Neutrophils can also be utilized as modes of transportation by these viruses to disseminate to other organs and tissues (Grundy et al., 1998). For example, neutrophils have been shown to transport virus from the site of infection to the bone marrow and promote a virus-specific CD8^+^ T cell response (Duffy et al., 2012).

Viruses are absolutely dependent on the host cell for replication, and therefore it is not surprising that they are capable of interfering with host signal transduction pathways that regulate cell fate. To this end, a number of viruses have been described to promote cell survival or induce rapid cell death. For example, respiratory syncytial virus (RSV) has been shown to activate nuclear factor-kappa B (NF-κB) and phosphatidylinositol 3-kinase (PI3K), which subsequently increases expression and stabilization of Mcl-1 protein and ultimately results in inhibition of neutrophil apoptosis (Lindemans et al., 2006). Human cytomegalovirus inhibits Fas-mediated apoptosis of neutrophils through a mechanism likely associated with the vICA protein that binds to procaspase-8 and prevents activation (Skaletskaya et al., 2001; Skarman et al., 2006). Influenza A has been reported to accelerate neutrophil apoptosis, and this process is associated with increased expression of Fas/FasL during infection (Colamussi et al., 1999). During viral infection, it is often difficult to distinguish between direct and indirect effects of the virus on neutrophil function and cell fate. For example, it is well known that virus-infected host cells can secrete cytokines and chemokines that trigger neutrophil influx and activation, both of which have an impact on neutrophil survival. Human immunodeficiency virus-1 (HIV-1) has direct and indirect effects on neutrophil function and survival. Several studies have demonstrated that spontaneous neutrophil apoptosis is increased in HIV-infected individuals (Pitrak et al., 1996; Casulli and Elbim, 2014). The mechanism for this process is incompletely determined, but may involve increased susceptibility to FAS-induced apoptosis, oxidative stress, and/or increased calpain activity (Salmen et al., 2004, 2007; Lichtner et al., 2006). The loss of neutrophils combined with impaired neutrophil function in HIV-infected individuals likely contributes to the known host susceptibility to secondary bacterial and/or fungal infections.

It is of note that several viruses have been shown to trigger neutrophil lysis and NET formation. Despite the relatively small size of most viruses, it has been suggested that the virus particles are physically constrained by NETs primarily due to differences in charge (Schonrich and Raftery, 2016). NET constituents such as α-defensins and MPO have been reported to directly inactivate some viruses (Hazrati et al., 2006; Saitoh et al., 2012). In contrast, deletion of peptidylarginine deiminase 4 (PAD-4), which is essential in histone deamination during NET formation, was shown to have no effect on viral load or neutrophil recruitment during infection, demonstrating a dispensable role of NETs during influenza infection (Hemmers et al., 2011). It is difficult to ascertain the role or significance of NETs during viral infection, likely because neutrophils have not been shown to be a primary target cell for replication of any virus and the role of PMNs in host defense against most viral pathogens is unclear. Notwithstanding, it is evident that NETs are deleterious to host tissue and contribute significantly to viral pathogenesis in pulmonary disease (Ramos-Casals et al., 2008; Narasaraju et al., 2011; Porto and Stein, 2016).

## Summary

The default endpoint for neutrophils during steady state conditions is spontaneous apoptosis and removal by other phagocytes. This default process is typically altered by the interaction with microbes or their products. The microbe-influenced fate of neutrophils can be beneficial or detrimental to the host, depending on the specific microbe and context of the interaction (e.g., low versus high bacterial burden). Possible outcomes for neutrophils following interaction with bacteria, fungi, parasites, and viruses are summarized in Figure 1. An outcome that results in the resolution of infection and the inflammatory response is favorable for host health, whereas the other potential outcomes lead to disease and are not beneficial to the host. In this model schematic, formation of NETs results from neutrophil lysis/necrosis—whether regulated or not—rather than a specialized form of death. It is noteworthy that NETs can form from viable cells, such as from release of mitochondrial DNA (Yousefi et al., 2009) or extrusion of nuclear DNA from viable neutrophils (Pilsczek et al., 2010), although these processes are not depicted here. It is also not clear if the neutrophil lifespan is altered significantly by these phenomena and more work in this area is needed.

## Author contributions

All authors listed, have made substantial, direct and intellectual contribution to the work, and approved it for publication.

### Conflict of interest statement

The authors declare that the research was conducted in the absence of any commercial or financial relationships that could be construed as a potential conflict of interest. The reviewer WMN and handling Editor declared their shared affiliation, and the handling Editor states that the process nevertheless met the standards of a fair and objective review.
